# The association of empowerment measures with maternal, child and family planning outcomes in Plateau State Nigeria by urban-rural residence

**DOI:** 10.1186/s12884-021-03659-y

**Published:** 2021-02-27

**Authors:** Kavita Singh, Ilene S. Speizer, Rashida-E Ijdi, Lisa M. Calhoun

**Affiliations:** 1 Department of Maternal and Child Health, University of North Carolina Gillings School of Global Public Health, Chapel Hill, NC USA; 2 Carolina Population Center, University of North Carolina at Chapel Hill, Chapel Hill, NC USA

**Keywords:** Women’s empowerment, Maternal health, Family planning, Decision-making, Nigeria

## Abstract

**Background:**

Nigeria is experiencing a high level of urbanization and urban poverty. Within Nigeria maternal and child health and family planning outcomes may differ by residence (capital city, urban/non-capital city and rural) as well as by measures of women’s empowerment and wealth. This paper presents a detailed analysis of maternal and child health and family planning outcomes in Plateau State, Nigeria.

**Methods:**

Data came from the 2017 Nigerian Urban Reproductive Health Initiative Sustainability Study. Multivariable logistic regression was used to study the associations between the key independent variables of residence, women’s empowerment and wealth with having a skilled birth attendant at childbirth and childhood preventative visits. The women’s empowerment variables included perceptions about household decision-making, financial decision-making, views on wife beating and having a prohibition, defined as a restriction on specific activities imposed by a woman’s husband. Multinomial regression was used to study the association of the same factors with the family planning outcome which had three categories – no use, traditional method use and modern method use. Regressions were also run separately for urban and rural populations.

**Results:**

Women in the capital city of Jos were significantly more likely to have a skilled birth attendant at childbirth, take a child to a preventative visit and use family planning than women in rural areas of Plateau State. Three of the four measures of empowerment (household decision-making, financial decision-making and having a prohibition) were significantly associated with the family planning outcome, while having a prohibition was negatively associated with having a skilled birth attendant at childbirth. In rural areas, women involved in financial decisions were significantly less likely to use a modern method compared to a traditional method. Wealth was a significant factor for all outcomes.

**Discussion:**

State-level analyses can provide valuable information to inform programs and policies at a local level. Efforts to improve use of maternal and child health and family planning services in Plateau state, Nigeria, should consider women’s empowerment, residence and poverty. Community education on the effectiveness of modern versus traditional methods and potential side effects of specific modern methods, may help women make informed decisions about contraception.

**Supplementary Information:**

The online version contains supplementary material available at 10.1186/s12884-021-03659-y.

## Background

With an estimated population of over 200 million, Nigeria is the most populous country in Africa and the seventh most populous country in the world [1]. Though Nigeria has seen improvements in maternal and under-five health, mortality remains high and contraceptive prevalence remains low. Maternal mortality was 917 maternal deaths per 100,000 live births in 2017 [2], and under-five mortality was 117 under five deaths per 1000 live births in 2019 [3]. Given Nigeria’s large population, this translates into 67,000 maternal deaths and 866,000 under-five deaths annually [2, 3]. In 2018, the total fertility rate (TFR) in Nigeria stood at 5.3 births per woman and the unmet need for family planning among currently married women was 18.9% [4].

Nigeria is a diverse country, divided into 36 states and home to individuals from 250 ethnic groups who speak over 500 languages. Nigeria has a system of decentralization whereby states have the authority to make some decisions regarding health care. In addition, given the size and diversity of the country, state level analysis of health outcomes is essential to inform programs and policies. Even within a state there can be large differentials in health outcomes by local government area and between urban versus rural populations. For example, of women with a live birth in the past 5 years, 61% of urban women compared to 26% of rural women delivered their baby in a health facility according to the 2018 Nigeria Demographic and Health Survey [4]. Furthermore, even within urban areas there are distinctions given that there is a high level of urban poverty About 54% of the urban population in Nigeria lives in slum settings according to the United Nations [5]. Nigeria is also experiencing considerable urbanization, which is changing many traditional and cultural social norms. In urban settings some of these changes result in couples living away from extended family and more women working outside the household for income than in rural settings [6–8]. Currently half of the Nigerian population lives in urban areas [9]. Given these demographic and cultural shifts, differences in health outcomes are more complex than a simple urban-rural distinction, and other factors, including women’s empowerment and poverty level, should be considered.

Women’s empowerment is a process of change whereby women who had previously been denied the ability to make important life choices are able, over time, to make those choices [10, 11]. Empowerment is often captured in household surveys through measures of decision-making or autonomy and views on social norms. Autonomy is often defined as the ability to participate in decision-making by virtue of information or resources and involves the ability to take actions based upon those decisions [12, 13]. Norms around gender equality are also important as an individual woman’s autonomy is often influenced by her social and cultural setting [14]. Attitudes toward the acceptability of wife beating under various circumstances is often studied as a social norm [14]. In addition, women may face restrictions or prohibitions on activities or movements due to norms or by family members. In some settings, including Northern Nigeria, women may need permission from their husband or other family members in order to leave their homes [15, 16].

Several studies have shown the influence of empowerment measures on maternal health, child health and family planning outcomes in Nigeria. In a study of six major cities from the northern and southern regions in Nigeria, Corroon and colleagues [6] found that women who scored higher on empowerment measures were more likely to deliver their baby in a health facility, have a skilled birth attendant at childbirth and use modern contraceptives. The empowerment-related measures used in that study were household decision-making, economic freedom, gender norms towards domestic violence and partner prohibitions and restrictions toward various behaviors. A state-level analysis of contraceptive use in Nigeria found that women living in states with higher percentages of women in the labor force, with secondary or higher education and with health-care related decision-making were significantly more likely to use modern contraceptives than women in states with lower levels of these empowerment-related indicators [17]. Alabi found that in Northern Nigeria an autonomy index (composed of measures on health decision-making, movement and an economic decision regarding large purchases) was associated with modern contraceptive use [16]. Further, a qualitative study from Kaduna found that many women indicated they would need their husband’s permission to use contraceptives [15]. Other studies from Nigeria have found significant associations between both household decision-making and views on domestic violence with childbirth in a health facility [18] and full immunization coverage for children [19]. Babalola (2009) [20] also found significant associations between household decision-making and children receiving the third dose of the diphtheria-pertussis-tetanus (DPT) vaccine in Northern Nigeria.

The objective of this paper is to present a detailed look at the influence of residence as well as measures of women’s empowerment on maternal and child health and family planning outcomes for Plateau State, Nigeria. Given the diversity of states in Nigeria, this sub-national level analysis will demonstrate the importance of such analyses for programs and policies. To the best of our knowledge such an in-depth examination of women’s empowerment and maternal, child and family planning outcomes has not been done for Plateau State.

## Methods

### Setting

Plateau State, which is located in the North Central Zone of Nigeria, has a population of over 3 million and is home to individuals belonging to over 40 ethno-linguistic groups [21]. Out of Nigeria’s 36 states, Plateau is the 12th largest in terms of geography and 31st in terms of population size [22]. Jos, the capital city, has a population of approximately 816,000 [23]. Forty-three percent of women in Plateau State delivered their baby with a skilled birth attendant, which is the same as for Nigeria overall [4]. In Plateau State, use of any method of family planning and use of a modern method among married women are 23 and 21%, respectively, compared to 17 and 12% for Nigeria overall [4]. Forty-eight percent of children in Plateau State received basic immunizations compared to 31% for Nigeria overall [4].

### Data

Data for this analysis come from a household survey conducted as part of the 2017 Nigerian Urban Reproductive Health Initiative (NURHI) Sustainability Study. The NURHI project was focused on improving supply and demand for family planning in six cities in Nigeria. The objective of the NURHI Sustainability Study was to evaluate the continuation of program impacts of the NURHI project in two of the original six cities: Ilorin and Kaduna. In Ilorin, project activities ended in 2015 and in Kaduna project activities continued. In addition, Jos, the capital city of Plateau State, was included as a comparison city where NURHI never had program activities. Representative, Plateau state-level data were collected to provide a broader perspective of the family planning situation in this state, prior to the launch of another program in this state. The survey included three strata in Plateau state: Jos, other urban areas, and rural areas.

Data for the NURHI Sustainability Study were collected using a multi-stage sampling approach. First, using information from the 2006 census undertaken by the National Population Commission, the team randomly selected enumeration areas (clusters) in all three cities (Ilorin, Kaduna, and Jos). For Plateau state, the study included 101 clusters, 56 of which were in Jos, 34 were rural, and 11 were other urban/non-capital. These clusters were selected from the full 2006 census sampling frame for the state and city. Listing and mapping was conducted in each selected cluster in July 2017. Fieldworkers updated census-level boundary maps and listed all households in selected clusters. From the list of households in selected clusters, the second stage sampling was to randomly select 33 households from each cluster to include in the survey. Upon receipt of consent from the household head to undertake a household survey, all women aged 15–49 years in the selected households were approached by female interviewers for their consent for the women’s survey. Interviewers completed the surveys using paper and pencil and asking the questions in the language that the respondent was most comfortable using (most often Hausa).

In total, 3163 households were included which resulted in 3653 women from Plateau state surveyed in 2017; this includes 2003 women from the capital city of Jos. In this paper we use data from 2151 married or cohabiting women ages 15–49 years from the three areas of Plateau State – Jos (*n* = 1046), other urban/non-capital (*n* = 254), and rural areas (*n* = 851).

### Outcome variables

Three outcome variables were studied in this analysis. A family planning outcome was created as a categorical variable with three categories- no use, traditional method use and modern method use. The traditional methods include the rhythm method, withdrawal and other methods such as the use of herbs and seeds. Modern methods include sterilization, implant, intra-uterine device (IUD), injectable, daily and emergency contraceptive pills, male and female condoms, breastfeeding/lactational amenorrhea, and standard days method (SDM). One maternal health outcome was studied: whether or not the last childbirth in the past 3 years was attended by a skilled birth attendant, defined as a doctor, nurse or midwife. The last outcome was focused on child health: whether or not a child under the age of two had a preventative health check in the last 3 months. Children under the age of two have several recommended wellness checks/immunizations at specific ages, which would make having a preventative check in the past 3 months a relevant outcome. The sample sizes for the latter two outcomes were smaller than 2151 because they only included married or cohabiting women with a birth in the past 3 years (*n* = 1158) or with a child under the age of two (*n* = 709).

### Independent variables

Key independent variables included four measures of women’s empowerment –perceptions about household decision-making, financial decision-making behaviors, prohibitions or restrictions by the husband on various activities and views on domestic violence. Respondents were asked, “In a couple, who do you think should have the greater say in each of the following decisions: the husband, the wife, or both equally?” The decisions asked about were a) Making large household purchases; b) Making small household purchases; c) Deciding when to visit family, friends, or relatives; and d) Deciding when and where to seek medical care for your own health. For each of the questions, women were coded one if they reported wife or both equally and zero otherwise. The decision-making attitude variable was created as an additive index ranging from zero to four. The measure of financial decision-making was based on a question, “Who decides how the money that your partner earns will be used?” Response options were mainly you, your partner or you and your partner jointly. Women who responded that they made the decision alone or jointly with their partner were classified as having a say in financial decisions. Women were also asked, “Sometimes in a marriage or a relationship, a man prohibits his wife from doing certain things. Does your husband prohibit you from: working outside the home, having visits from other people, visiting friends, visiting family, and using a mobile phone?” We created a prohibition variable, which was coded as zero if a woman responded that she did not face any prohibitions (i.e., no to all questions) and was coded one if she responded yes to facing any of the five prohibitions. Also included was a gender norms measure asking about women’s views on domestic violence. This was coded as zero if a woman responded that each of seven circumstances did not warrant wife beating and was coded one if she responded that any one circumstance warranted wife beating. The circumstances were 1) goes out without telling husband, 2) neglects the house or the children, 3) argues with husband, 4) refuses to have sex, 5) cooks the food improperly, 6) suspects her of being unfaithful and 7) refuses to have another child.

Other key independent variables included residence within Plateau State (Jos, urban/ non-capital city and rural areas) and wealth. The measure of wealth was created based on household characteristics and assets available to households. In order to create wealth indices reflective of the circumstances in the different places of residence (Jos, urban/non-capital city, and rural), a separate principal components analysis was undertaken for each location. Wealth was categorized into quintiles in each location. Then an overall wealth variable was created by combining the quintiles across the three locations. For example, the lowest wealth quintile was created by combining the lowest wealth category for each of the three locations. The same was done for the four other categories of wealth. This approach was used to avoid classifying most women from Jos as rich and most women from rural areas as poor. Our wealth variable is, thus, relative to the woman’s place of residence.

Also included in the analyses were several control variables. Maternal age was categorized into five age groups – 15–19, 20–24, 25–29, 30–34 and 35 and over. Age was measured as a categorical variable because maternal health use and family planning needs may differ for older or younger women or among women in different age categories. Two different categorizations for parity were created – one for the family planning outcomes and one for the childbirth with a skilled birth attendant and child health outcomes. The reason for this was that nulliparous women could not be included in the sample for the latter two outcomes. The first parity variable had the categories of 0–1, 2, 3–4 and 5 and over. The second parity variable had the categories of 1, 2–3, 4–5 and 6 and over. The categorizations were also slightly different because women who have never given birth or have only one child may have different family planning needs than women with 2 or more children. For the skilled childbirth outcome, it is important to separate out first births because they, along with higher order births, are often considered riskier. The different needs and risks for women at low and high parities are the reasons why the two parity measures were categorical variables. The maternal education variable included three levels – none and non-standard, primary, and secondary and higher. The classification for religion was Christian and Muslim/other.

### Statistical analyses

Descriptive analyses were employed to provide insight into the characteristics of the sample of women. Comparisons for the independent variables were made by residence, and chi-square tests were used to test for statistical significance. Multivariable logistic regression analyses were used to understand whether the measures of empowerment, location and wealth status were significantly associated with the maternal and the child health outcome, after controlling for demographic factors. Multivariable multinomial regression was used to study family planning since there were three categories – no use, traditional method use and modern method use. The analyses took into account the cluster survey design, and sampling weights specific to Plateau state were applied for the descriptive analyses. Analyses were run for the full sample and also stratified by location. Sampling weights were calculated based on the size of the population in each of the study areas and adjusted for non-response. Because the parent study was meant to compare the women from Jos to women from two other cities (Kaduna and Ilorin), we over-sampled women from Jos. Weights are used to adjust the sample from Plateau state to represent the actual distribution of the population.

## Results

Table 1 includes a description of each of the independent variables based on the unweighted sample of 2151 women. Data are presented for the total sample of women married or cohabiting (i.e. in union) and also stratified by location – Jos, urban/non-capital and rural areas. Sixteen percent of the weighted sample lived in Jos, followed by 27% in other urban areas and 57% in rural areas of Plateau state. In terms of age, 42% of the sample was in the age range 35 to 49, and 21% was in the age range 25–29. Nineteen percent of women were nulliparous or of parity one, while 40% had five or more live births. There were significantly more nulliparous women and women of parity one, and fewer women with higher order births in Jos compared to the other urban and rural areas. Sixty-six percent of the sample was Christian, while 34% were Muslim or another religion. Forty-five percent of the women had some secondary education or higher. The differences in education were significant by location with 73% of women in the Jos sample having secondary or higher education; women from other urban areas also were more educated than their rural counterparts (56 and 32% respectively). As expected, there was a fairly even distribution across the wealth quintiles with between 19 and 22% of women in each category. As mentioned, location specific wealth information was used to create the overall wealth variable.

**Table 1:** Descriptive Characteristics of the Sample (Survey Weighted Percentages)

Sociodemographic Factors	Full Sample (%)	Jos (%)	Urban/non-Capital City (%)	Rural (%)
**Location**				
Jos	16.3	NA	NA	NA
Plateau State Urban	26.7	NA	NA	NA
Plateau State Rural	57.0	NA	NA	NA
**Age +**				
15–19	5.4	1.7	5.4	6.4
20–24	14.9	13.0	11.6	17.0
25–29	20.6	24.2	21.1	19.3
30–34	17.5	21.4	18.9	15.7
35–49	41.7	39.7	43.0	41.6
Parity **				
0–1	19.3	24.5	16.8	19.0
2	13.6	17.8	13.1	12.6
3–4	27.4	30.3	34.7	23.1
5+	39.8	27.4	35.4	45.3
Education **				
None/nonformal	24.7	11.1	19.0	31.2
Primary	30.2	16.4	24.5	36.8
Secondary and Higher	45.1	72.5	56.4	32.0
Wealth quintile**				
Lowest	19.4	4.0	28.1	19.7
Second	18.6	10.9	22.8	18.9
Middle	22.0	24.7	19.9	22.3
Fourth	20.3	24.9	17.5	20.4
Highest	19.6	35.5	11.7	18.7
**Religion**				
Christian/Catholic	65.7	54.6	70.5	66.7
Muslim/Other	34.3	45.4	29.5	33.3
**Decision-making Involvement**				
0 of 4 decisions	14.5	13.7	15.6	14.3
1 of 4 decisions	14.9	14.7	12.3	16.2
2 of 4 decisions	21.7	26.5	19.7	21.4
3 of 4 decisions	21.4	23.9	22.5	20.1
4 of 4 decisions	27.4	21.2	29.9	28.1
**Financial Decision-making**				
No	51.6	54.6	44.9	53.9
Yes	48.4	45.4	55.1	46.1
Prohibitions **				
No	91.2	84.0	94.8	91.6
Yes	8.8	16.0	5.2	8.4
Wife Beating Acceptable ***				
No	58.5	87.4	65.1	47.2
Yes	41.4	12.6	34.9	52.8

The significance test is compared across the places of residence

The unweighted counts were 2151 for full sample, 1046 for Jos, 254 for urban/non-capital city, and 851 for rural

The weighted counts were 2300 for full sample, 375 for Jos, 615 for urban/non-capital city, and 1310 for rural

Some of the n’s may differ slightly due to small amounts of missing data

+*p* < 0.10 *p < 0.05 **p < 0.01 ***p < 0.001

Regarding the empowerment measures, most of the sample of women believed that women should be involved in two or more household decisions. The percentages were 22, 21 and 27% for two, three and four household decisions, respectively. In terms of financial decision-making, 48% of the sample indicated that they were involved in financial decisions, and there were no significant differences by location. The majority of women (91%) indicated they did not have any prohibition or restrictions. Differences by location were significant with a greater percentage of women in Jos indicating they had experienced a prohibition (16%). Forty-one percent of women reported that domestic violence was acceptable for at least one of the seven listed circumstances. Differences by location were significant with 53% of women in rural areas, but only 13% in Jos, indicating that wife-beating was acceptable in one or more circumstances.

Table 2 presents the outcome variables by location – Jos, other urban and rural. Sixty-seven percent of women in the sample were not using family planning, 6% were using a traditional method and 27% were using a modern method. Differences by residence were not significant, though interestingly traditional use was highest in Jos with 9.8% of women using a traditional method. Forty-four percent of women had a skilled attendant at childbirth, and there were significant differences by location. Sixty-five percent of women in Jos, followed by 58% of women in other urban areas and 33% of women in rural areas of Plateau state had a skilled attendant at childbirth. There were also significant differences by location for the use of preventative child health services in the past 3 months. The percentage was highest in other urban areas at 64%, followed by 59% in Jos and 43% in rural areas with 51% overall.

**Table 2:** Descriptive Characteristics of the outcome variables for women married or cohabiting in Plateau State, Nigeria 2017 (Survey Weighted Percentages and Unweighted Counts)

Outcome Variables	Full Sample (%)	Jos (%)	Other Urban (%)	Rural (%)
**Family Planning Method**				
None	67.4	60.5	64.3	70.8
Traditional methods	5.9	9.8	7.2	4.2
Modern methods	26.7	20.7	28.5	25.0
Skilled Attendant at Childbirth ***				
No	55.8	35.5	41.9	66.7
Yes	44.2	64.5	58.1	33.3
Child Wellness Check in the Past Three Months ***				
No	48.8	41.5	36.3	57.1
Yes	51.2	58.5	63.7	42.9

Note: The significance test is compared across the places of residence

The unweighted counts were 2151 for full sample, 1046 for Jos, 254 for urban/non-capital city, and 851 for rural

The weighted counts were 2300 for full sample, 375 for Jos, 615 for urban/non-capital city, and 1310 for rural

Some of the n’s may differ slightly due to small amounts of missing data

+*p* < 0.10 *p < 0.05 **p < 0.01 ***p < 0.0

Tables 3, 4, 5 and 6 include the results of the multivariable analysis of the three outcomes for the full sample and also stratified by urban (Jos and other urban areas combined) and rural areas. Jos and other urban areas were combined to increase the sample size; however, the analysis included a variable indicating residence in Jos or other urban areas.

**Table 3:** Relative risk ratios (RRR) and 95% confidence intervals from multinomial logistic regression model for use of family planning methods and empowerment measures and location after controlling for sociodemographic factors among women in union from Plateau State, Nigeria, 2017

Sociodemographic Factors	Traditional Method vs. Non-User			Modern Method vs. Non-User		
	Full Sample	Urban Sample	Rural Sample	Full Sample	Urban Sample	Rural Sample
	RRR (CI)	RRR (CI)	RRR (CI)	RRR (CI)	RRR (CI)	RRR (CI)
**Age group** (Ref: 25–29)						
15–19	1.36 (0.31–5.79)	0.73 (0.07–7.29)	2.63 (0.33–20.96)	1.41 (0.63–3.17)	1.96 (0.66–5.82)	1.10 (0.32–3.75)
20–24	1.71 (0.86–3.40)	2.11+ (0.98–4.55)	0.86 (0.21–3.46)	1.50+ (0.99–2.27)	1.35 (0.72–2.53)	1.64+ (0.95–2.84)
30–34	1.14 (0.64–2.00)	1.16 (0.64–2.09)	0.95 (0.14–6.27)	1.10 (0.75–1.61)	1.03 (0.65–1.64)	1.00 (0.51–1.95)
35+	0.70 (0.39–1.28)	0.64 (0.33–1.26)	0.91 (0.23–3.50)	0.52*** (0.36–0.73)	0.49** (0.33–0.74)	0.48* (0.24–0.95)
**Parity** (Ref: 0–1)						
2	2.76** (1.41–5.40)	2.61* (1.23–5.55)	4.77+ (0.96–23.67)	3.43*** (2.34–5.03)	3.65*** (2.25–5.93)	3.26** (1.63–6.50)
3–4	2.65** (1.35–5.21)	2.83** (1.35–5.95)	2.85 (0.43–18.68)	5.92*** (4.05–8.66)	7.02*** (4.45–11.05)	4.93*** (2.61–9.31)
5+	3.47** (1.63–7.39)	2.86* (1.16–7.06)	6.69* (1.04–42.97)	10.24*** (6.48–16.18)	9.99*** (5.54–18.01)	13.5*** (6.05–30.19)
**Education level** (Ref: None/Non-standard)						
Primary	3.51** (1.43–8.60)	3.42* (1.25–9.33)	3.48 (0.54–22.30)	1.38 (0.88–2.19)	0.86 (0.47–1.58)	2.41** (1.29–4.49)
Secondary or higher	3.90** (1.71–8.90)	3.23* (1.26, 8.30)	5.71+ (0.81–40.24)	1.70* (1.11–2.59)	1.23 (0.68–2.22)	2.89** (1.49–5.61)
**Wealth quintile** (Ref: Middle)						
Lowest	0.30* (0.12–0.76)	0.30* (0.09–0.95)	0.30 (0.05–1.79)	0.81 (0.51–1.26)	0.83 (0.47–1.45)	0.87 (0.45–1.67)
Second	0.30** (0.14–0.68)	0.27** (0.10–0.69)	0.44 (0.09–2.13)	0.70+ (0.48–1.02)	0.64+ (0.40–1.04)	0.81 (0.43–1.52)
Fourth	0.75 (0.46–1.23)	0.65 (0.38–1.11)	0.87 (0.22–3.34)	0.98 (0.68–1.40)	0.83 (0.50–1.37)	1.13 (0.69–1.85)
Highest	1.02 (0.59–1.78)	0.93 (0.50–1.72)	1.05 (0.28–3.85)	1.06 (0.76–1.48)	0.83 (0.55–1.24)	1.58 (0.83–3.01)
**Religion** (Ref: Christian/Catholic)						
Muslim/Other	0.97 (0.60–1.58)	0.95 (0.54–1.69)	1.10 (0.34–3.52)	0.24*** (0.15, 0.39)	0.18*** (0.12–0.28)	0.47 (0.19–1.18)
**Location** (Ref: Jos)						
Other Urban	0.77 (0.37–1.62)	0.81 (0.38–1.73)	NA	0.68+ (0.43–1.05)	0.60* (0.38–0.95)	NA
Rural	0.40** (0.23–0.68)	NA	NA	0.53** (0.36–0.80)	NA	NA
**Gender Equality Measures**						
**Household Decision Making**	1.10 (−)	1.17 (−)	0.93 (−)	1.16** (−)	1.14* (−)	1.18* (−)
**Financial Decision-making**	2.13** (1.31–3.48)	1.84* (1.04–3.27)	3.67* (1.19–11.26)	1.13 (0.89–1.43)	1.13 (0.82–1.55)	1.04 (0.72–1.50)
**Prohibitions**	0.55+ (0.30–1.02)	0.52+ (0.27–1.00)	0.68 (0.08–5.56)	0.65+ (0.41–1.04)	0.49* (0.29–0.85)	1.50 (0.63–3.59)
**Wife-beating Acceptable**	0.86 (0.54–1.37)	0.63 (0.31–1.28)	1.23 (0.55–2.74)	1.07 (0.81–1.40)	0.99 (0.68–1.43)	1.07 (0.71–1.62)

The significance test is compared across the places of residence

The unweighted counts were 1981 for full sample, 1192 for urban, and 789 for rural areas

+*p* < 0.10 *p < 0.05 **p < 0.01 ***p < 0.001

**Table 4:** Relative risk ratios (RRR) and 95% confidence intervals from multinomial logistic regression model for use of family planning methods and empowerment measures and location after controlling for sociodemographic factors among women in union from Plateau State, Nigeria, 2017

Sociodemographic Factors	Modern methods vs. Traditional methods		
	Full Sample	Urban Sample	Rural Sample
	RRR (CI)	RRR (CI)	RRR (CI)
**Age group** (Ref: 25–29)			
15–19	1.04 (0.21–5.00)	2.66 (0.27–26.34)	0.41 (0.03–4.94)
20–24	0.87 (0.41–1.82)	0.64 (0.27–1.46)	1.90 (0.44–8.26)
30–34	0.96 (0.55–1.68)	0.89 (0.48–1.62)	1.05 (0.19–5.78)
35+	1.73 (0.407–1.32)	0.76 (0.40–1.46)	0.52 (1.15–1.78)
**Parity** (Ref: 0–1)			
2	1.24 (0.63–2.44)	1.39 (0.67–2.92)	0.68 (0.11–4.11)
3–4	2.23* (1.10–4.50)	2.47* (1.16–5.26)	1.73 (0.21–13.85)
5+	2.94** (1.35–6.38)	3.48** (1.39–8.71)	2.02 (0.24–16.56)
**Education level** (Ref: None/Non-standard)			
Primary	0.39+ (0.15–1.02)	0.25** (0.09–0.68)	0.69 (0.09–4.81)
Secondary or higher	0.43+ (0.17–1.05)	0.38+ (0.14–1.00)	0.50 (0.06–3.76)
**Wealth quintile** (Ref: Middle)			
Lowest	2.63+ (1.00–6.92)	2.69+ (0.89–8.08)	2.82 (0.46–17.31)
Second	2.27* (1.01–5.08)	2.39+ (0.90–6.32)	1.83 (0.38–8.81)
Fourth	1.29 (0.77–2.18)	1.27 (0.71–2.27)	1.29 (0.34–4.85)
Highest	1.03 (0.59–1.82)	0.89 (0.48–1.64)	1.50 (0.36–6.10)
**Religion** (Ref: Christian/Catholic)			
Muslim/Other	0.25*** (0.13–0.47)	0.19*** (0.09–0.38)	0.43 (0.10–1.76)
**Location** (Ref: Jos)			
Other Urban	0.87 (0.44–1.72)	0.74 (0.37–1.50)	NA
Rural	1.34 (0.78–2.28)	NA	NA
**Gender Equality Measures**			
**Household Decision Making**	1.05 (0.87–1.27)	0.97 (0.77–1.21)	1.26 (0.86–1.86)
**Financial Decision-making**	0.53* (0.32–0.87)	0.61 (0.33–1.12)	0.28* (0.09–0.85)
**Prohibitions**	1.17 (0.56–2.42)	0.94 (0.42–2.11)	2.19 (0.23–20.72)
**Wife-beating Acceptable**	1.23 (0.76–1.99)	1.54 (0.77–3.09)	0.87 (0.37–2.05)

The significance test is compared across the places of residence

The unweighted counts were 1981 for full sample, 1192 for urban, and 789 for rural areas

+*p* < 0.10 *p < 0.05 **p < 0.01 ***p < 0.001

**Table 5:** Odds ratios and 95% confidence intervals from logistic regression models for childbirth with a skilled attendant and empowerment measures and location after controlling for sociodemographic factors

Sociodemographic Factors	Full Sample	Urban Sample	Rural Sample
	OR (CI)	OR (CI)	OR (CI)
**Age group** (Ref: 25–29)			
15–19	0.41^+^ (0.16–1.07)	0.49 (0.13–1.87)	0.35 (0.09–1.34)
20–24	0.71 (0.44–1.17)	0.73 (0.38–1.39)	0.69 (0.33–1.45)
30–34	0.89 (0.61–1.30)	1.14 (0.66–1.97)	0.55 ^+^ (0.29–1.06)
35+	1.05 (0.67–1.64)	1.38 (0.77–2.46)	0.65 (0.28–1.53)
**Parity** (Ref: 0–1)			
2	0.46** (0.29–0.73)	0.38 ** (0.20–0.71)	0.58 (0.29–1.17)
3–4	0.27 *** (0.16–0.44)	0.27 *** (0.14–0.52)	0.30 ** (0.14–0.65)
5+	0.27 *** (0.15–0.50)	0.22 *** (0.10–0.49)	0.39 * (0.16–0.97)
**Education level** (Ref: None/Non-standard)			
Primary	0.54 * (0.33–0.86)	0.52 ^+^ (0.27–1.02)	0.64 (0.29–1.39)
Secondary or higher	0.87 (0.54–1.40)	0.89 (0.47–1.70)	0.90 (0.40–2.00)
**Wealth quintile** (Ref: Middle)			
Lowest	0.61 ^+^ (0.35–1.04)	0.72 (0.36–1.48)	0.48 ^+^ (0.21–1.10)
Second	0.50 ** (0.31–0.83)	0.40 * (0.19–0.84)	0.63 (0.31–1.31)
Fourth	1.39 (0.87–2.22)	1.70 (0.85–3.39)	0.98 (0.50–1.90)
Highest	2.03 ** (1.28–3.23)	1.82 * (1.01–3.29)	2.33 * (1.11–4.91)
**Religion** (Ref: Christian/Catholic)			
Muslim/Other	0.18*** (0.12–0.28)	0.13*** (0.08–0.20)	0.26** (0.11–0.62)
**Location** (Ref: Jos)			
Other Urban	0.74 (0.44–1.27)	NA	NA
Rural	0.22 *** (0.13–0.35)	NA	NA
**Jos** (Ref: No)			
Yes	NA	1.28 (0.77–2.14)	NA
**Gender Equality Measures**			
**Household Decision Making**	0.98 (0.86–1.12)	1.00 (0.81–1.23)	0.94 (0.79–1.12)
**Financial Decision-making**	1.14 (0.83–1.56)	1.15 (0.79–1.67)	1.10 (0.63–1.94)
**Prohibitions**	0.57 ** (0.39–0.830	0.62* (0.41–0.94)	0.53 (0.19–1.46)
**Wife-beating Acceptable**	1.38^+^ (0.96–2.00)	0.82 (0.51–1.32)	1.94 ** (1.23–3.06)

The significance test is compared across the places of residence

The unweighted counts were 1058 for full sample, 608for urban, and 450 for rural areas

+*p* < 0.10 *p < 0.05 **p < 0.01 ***p < 0.001

**Table 6:** Odds Ratios and 95% confidence intervals from logistic regression models for child health preventative visit and empowerment measures and location after controlling for sociodemographic factors

Sociodemographic Factors	Full Sample	Urban Sample	Rural Sample
	OR (CI)	OR (CI)	OR (CI)
**Age group** (Ref: 25–29)			
15–19	1.22 (0.48–3.08)	0.85 (0.16–4.49)	1.28 (0.30–5.39)
20–24	0.63^+^ (0.39–1.00)	0.72 (0.43–1.22)	0.41^+^ (0.16–1.09)
30–34	1.07 (0.73–1.57)	0.95 (0.62–1.46)	1.38 (0.67–2.83)
35+	0.72 (0.42–1.22)	0.58 ^+^ (0.31–1.07)	1.20 (0.42–3.43)
**Parity** (Ref: 0–1)			
2	0.56 * (0.36–0.89)	0.63 ^+^ (0.36–1.09)	0.51 (0.21–1.26)
3–4	0.86 (0.49–1.51)	1.07 (0.57–0 2.01)	0.48 (0.12–1.91)
5+	0.72 (0.42–1.22)	0.97 (0.41–2.29)	0.54 (0.14–2.06)
**Education level** (Ref: None/Non-standard)			
Primary	1.89 * (1.05–3.40)	1.75 (0.71–4.33)	1.65 (0.68–4.02)
Secondary or higher	2.01* (1.08–3.72)	1.78 (0.86–3.71)	1.52 (0.48–4.82)
**Wealth quintile** (Ref: Middle)			
Lowest	0.54^+^ (0.27–1.09)	0.59 (0.26–1.33)	0.73 (0.22–2.39)
Second	0.79 (0.42–1.47)	0.66 (0.30–1.48)	1.02 (0.36–2.84)
Fourth	1.64^+^ (0.92–2.93)	1.11 (0.58–2.15)	3.23* (1.24–8.43)
Highest	0.95 (0.60–1.51)	0.77 (0.45–1.32)	1.39 (0.62–3.14)
**Religion** (Ref: Christian/Catholic)			
Muslim/Other	0.95 (0.59–1.52)	1.25 (0.71–2.19)	0.54 (0.19–1.57)
**Location** (Ref: Jos)			
Other Urban	1.40 (0.79–2.47)	NA	NA
Rural	0.66 ^+^ (0.41–1.06)	NA	NA
**Jos** (Ref: No)			
Yes	NA	0.70 (0.37–1.33)	NA
**Gender Equality Measures**			
**Household Decision Making**	0.97 (0.84–1.12)	1.06 (0.86–1.30)	0.88 (0.70–1.09)
**Financial Decision-making**	1.26 (0.89–1.78)	1.11 (0.70–1.76)	1.65 (0.88–3.10)
**Prohibitions**	1.17 (0.63–2.18)	0.83 (0.44–1.58)	2.12 (0.47–9.51)
**Wife-beating Acceptable**	1.16 (0.75–1.79)	0.93 (0.47–1.83)	1.41 (0.75–2.68)

The significance test is compared across the places of residence

The unweighted counts were 649 for full sample, 404for urban, and 245 for rural areas

+*p* < 0.10 *p < 0.05 **p < 0.01 ***p < 0.001

Table 3 presents results of the multinomial logistic regression of the use of a traditional method (vs. non-use) and a modern method (vs. non-use) among women married or in union in Plateau state. The results are presented for the full sample and then for urban and rural sub-samples. In the full sample, for comparison of ‘traditional method users’ versus ‘non-users’, the education level variable was significant. Women who had attained higher levels of education had higher relative risk of traditional method use than non-use (for primary completed RRR: 3.51 CI:1.43, 8.60, *p* < 0.01; for secondary or higher level of education completed (RRR: 3.90 CI: 1.71, 8.90, *p* < 0.01) compared to those with no education. The probability of using a traditional method compared to using no methods was 70% lower among women in the lower wealth quintiles compared to women in the middle wealth quintile; no difference was found between the three highest wealth groups. Two of the four measures of women’s empowerment were significant. Women who participated in financial decision making were more than twice as likely to use traditional method than to be non-user than their counter parts who did not participate in financial decision-making (RRR: 2.13, CI: 1.31, 3.48, *p* < 0.01). Women with a prohibition were 45% less likely to use traditional methods than be non-users, than women without prohibitions, but this finding was only significant at *p* < 0.10 (RRR:0.55, CI: 0.30, 1.02). Also significant was parity; women of higher parity were significantly more likely to use a traditional method than be non-users compared to their counterparts. No difference was found by religion and location of residence in this full model. In the analyses stratified by place of residence, financial decision-making was significant in both the urban and rural models, while having prohibitions was significant in the urban but not rural model.

Table 3 also shows results of the comparison between modern method users and non-users. Women in rural areas of Plateau state were significantly less likely to use any modern method (and more likely to be non-users) than women in Jos (RRR: 0.53, CI: 0.36, 0.80, *p* < 0.01). Women who scored higher on the decision-making index were significantly more likely to use a modern method than women involved in fewer decisions (RRR:1.16, CI: 1.05, 1.28, *p* < 0.01). Also significant were education, parity, and religion in the same directions as shown for the comparison between traditional method use and no use, such as higher education, Christianity, and higher parity are associated with being a modern method user. In the stratified models, household decision-making was significantly associated with the use of any modern method for both the urban and rural sample, and having prohibitions was significant in a negative direction in the urban model such that those who reported prohibitions were less likely to use a modern method than those without prohibitions (RRR: 0.49, CI: 0.29, 0.85, *p* < 0.05).

Also, in order to make comparisons between modern method use and traditional method use we also ran multinomial regression for the family planning outcome with traditional method use as the reference category. These results are presented in Table 4**.** Some notable findings were that the residence variable was not significant, and that more educated women were significantly less likely to use modern methods (compared to traditional methods) than women with no education. Women in the lower two wealth quintiles were more likely to use modern than traditional methods compared to women in the middle quintile. This finding was significant in the full sample and the urban sample. Women who were involved in financial decision-making were less likely to use a modern method (compared to a traditional method) then women who were not involved in financial decisions. The later finding was significant for both the full sample (RRR:0.53, CI:0.32, 0.87, *p* < 0.05) and the rural sample (RRR:0.28, CI:0.09, 0.85, *p* < 0.05).

Results for the analysis with the outcome of childbirth with a skilled birth attendant are displayed in Table 5**.** Women living in rural areas were 78% less likely to have a skilled delivery than women in Jos (OR: 0.22, CI: 0.13, 0.35, *p* < 0.000). Women with a prohibition were 43% less likely to have a skilled attendant at delivery than women with no prohibitions (OR:0.57, CI:0.39, 0.83, *p* < 0.005). Belief that domestic violence is acceptable in at least one situation was positive and marginally significant (OR:1.38, CI:0.96, 2.00, *p* < 0.070); this was an unexpected result. Women in the highest wealth quintile were significantly more likely to have childbirth with a skilled birth attendant than women in the middle wealth quintile, while women in the lowest two wealth quintiles were significantly less likely to have a childbirth with a skilled delivery attendant. Other significant factors included religion and parity, with women of the Muslim or other faiths and women of higher parity being less likely to have a skilled delivery than their counterparts. Unexpectedly women with primary education were less likely to have childbirth with a skilled birth attendant than women with no education or non-standard education. In the stratified models, having prohibitions was negative and significant only in the urban model, while the belief that wife-beating is acceptable was positive and significant only in the rural model.

Table 6 contains multivariate findings for the child health outcome. There were neither significant findings for the empowerment measures in the full sample nor in the stratified models. In the full model, children living in rural areas were 34% less likely to have had a preventative check than children in Jos (OR:0.66, CI: 0.41, 1.06, *p* < 0.10). There were some marginally significant findings for the wealth variable, such that children from poorer families were less likely to have a preventative check in the past 3 months. The children of more educated mothers were significantly more likely to have a preventative check. Findings for age and parity were not consistent and may reflect collinearity or small sample sizes. In the stratified models, few significant differences were found; this might reflect small sample sizes as reflected in the large confidence intervals.

Given the interest in understanding the difference between capital cities and other urban areas, interaction terms between Jos and the empowerment variables were tested for the urban sample. Interaction terms were only significant for the family planning outcome. Significant interactions were found for the comparison between traditional versus non-use such that the interaction for financial decision-making and Jos was positive and significant and the one for prohibitions and Jos was negative and significant. In the comparison of modern versus non-use, the only significant interaction (*p* < 0.10) was for the wife beating attitude and Jos which was negative. Finally, in the comparison between modern vs. traditional methods, interactions for financial decision-making and wife beating attitudes that had negative and significant interactions with Jos. (These results are in the Supplementary File 1).

## Discussion

Efforts to improve maternal and child health outcomes and increase family planning access and use in Nigeria must take into consideration the diversity of the country. Given the country’s large population and rich and varying cultures, state-level analyses can enable local-level program planners and policymakers to have the information needed to make decisions and allocate resources. Place of residence, wealth and women’s empowerment are all important measures which influence a women’s ability to access services. We presented detailed analyses of key maternal and child health and family planning outcomes specifically for Plateau State, Nigeria.

Results from our analyses indicated that women in the capital city of Jos had higher use of services than women in rural areas for all three outcomes. There are many differences in urban and rural areas in terms of socio-economic development, access to health services and women’s empowerment. Focusing on urban and rural residence alone, however, is not enough. A high proportion (44%) of Nigeria’s overall population is estimated to live in extreme poverty, defined as living on less than $1.90 per day [24]. Projections suggest that this figure will rise over time if interventions are not undertaken. Interestingly Cuaresma and colleagues (2018) [25] highlight the difficulty in coming up with one national poverty estimate for Nigeria given the diversity of the country. A strength of state-level analysis is the ability to focus on appropriate measures of wealth for specific settings. In our study, location-specific wealth variables were combined to create an overall wealth variable. This takes into consideration differing concepts of wealth in urban versus rural populations. For example, land and livestock ownership are important measures of wealth in rural, but not urban settings. In our study, wealth status was significant for all of the outcomes. Addressing poverty, along with providing educational opportunities, can be seen as steps towards enabling empowerment. Women without an education and living in extreme poverty in their setting may face limited choices in terms of life decisions and healthcare seeking [19, 26].

Studies in Nigeria have indicated that women who have more say in decisions and finances may be better able to access services for themselves and their children [6, 17–19]. The United Nations Population Fund (UNFPA) also states that giving women access to voluntary family planning is a means to combat poverty [27]. Given Nigeria’s rapid urbanization, there may be considerable changes in traditional norms which lead to changes in women’s empowerment and views on gender norms [6, 7]. These changes should be studied over time using longitudinal data at the individual or community levels.

In our study, three of the four gender equality measures were significantly associated with the use of any family planning method (either traditional or modern methods). Overall women with higher decision-making may be more comfortable talking about family planning with their spouses, and women with fewer prohibitions may have greater agency in seeking out family planning services when needed [6, 28].

For the childbirth with a skilled birth attendant outcome having a prohibition was significant in a negative direction, and the belief that wife-beating is acceptable was positive and marginally significant. The later was an unexpected finding but is not unprecedented as other studies have found a positive association between domestic violence attitudes and health outcomes. For example, earlier studies in the Philippines and six Sub-Saharan African countries (Cameroon, Kenya, Malawi, Rwanda, Uganda, and Zimbabwe) found a significant, positive relationship between domestic violence attitudes and modern contraceptive use [29, 30]. Also, a multi-country analysis of DHS data found that in seven countries (Bangladesh, Bolivia, the Dominican Republic, Haiti, Kenya, Malawi, and Zimbabwe), ever-use of contraception was positively associated with domestic violence [31]. More work, particularly qualitative, would be needed to further understand the marginally significant association between beliefs surrounding wife beating and skilled delivery in Nigeria. Women with fewer prohibitions on their activities may be better able to leave their homes to seek maternal health services such as antenatal care and a facility delivery, rather than having to wait to seek permission from spouses and other family members [15, 16].

Interestingly women with more financial decision-making and who were more educated preferred traditional over modern methods. A study in southwestern Nigeria has found that many women fear side effects of modern contraceptives and may find modern methods more difficult to access than traditional methods [32]. Another study of women attending primary health centers in Kano, Nigeria found women were more knowledgeable about traditional methods than modern methods [33]. According to the 2018 Nigeria DHS among currently married women ages 15–49 years, traditional method use is more common among urban women (8.1%) compared to rural women (2.2%). A similar finding was apparent for wealth quintiles with 10.7% of women in the highest wealth quintile using a traditional method compared to 0.7% of women in the lowest wealth quintile [4]. A survey of women in the capital city of Nigeria Lagos, also found traditional use to be highest among the wealthiest and most educated women [34]. Perhaps wealthy and educated women in urban areas of Nigeria are choosing to use traditional methods to avoid some of the common side effects of modern methods. It is important to educate all women on the effectiveness of modern versus traditional methods and what side effects may be possible. It is also essential to educate women on how common side effects are and what can be done to mitigate them.

The lack of significance of the empowerment measures with the child health outcome could be due to easier availability of preventative health services, compared to skilled delivery and family planning services. Preventative services for children can be provided at a range of facility types from health posts to health clinics to hospitals and are commonly provided by community health workers. For example, the Integrated Management of Childhood and Neonatal Illness (IMNCI) program, which is commonly used in low and middle income countries, includes training community health workers on providing both preventative and curative treatment for children [35]. In addition, the topic of child health may be a less sensitive topic than family planning for many couples.

The stratified models revealed that some of the empowerment measures were significant in both urban and rural areas, while some were only significant in one area. Three of the four measures of empowerment were significantly associated with the use of family planning (traditional or modern) in the urban models, while household and financial decision-making were significant in the rural models. For the skilled delivery outcome, prohibitions were only significant for the urban model, while the belief that wife beating is acceptable was only significance in the rural model. The stratified models offer more insight into nuanced issues to consider for program planning in urban versus rural areas. It is evident, however, that both household and financial decision-making are important for family planning use in both urban and rural areas.

There are some limitations to this study. The relatively small sample size of Jos and the other urban areas made it difficult to find significant differences between the two. We did test some interactions for the urban models and found mixed results. Capital cities may be different than other urban areas in a state and it would be important to explore such differences in more detail in future studies. Low power may have also been an issue in the stratified analysis for the family planning outcome. Other limitations are the inability to study other key maternal and child health outcomes, such as antenatal care and childhood immunizations due to small sample sizes. Our measure of child health is not a globally standardized indicator, and though children ages one to two do have recommended preventative visits they do not necessarily need to be every 3 months. In addition, our household decision-making measure is based on perception and not on actions. This study also only looks at associations and not causation. Future research would be helpful in uncovering whether specific measures of women’s empowerment are more relevant in urban versus rural settings, and whether additional measures should be included in household surveys. For example, perhaps women in urban areas face fewer restrictions on mobility, but more restrictions in other aspects unique to urban life like driving or types of work outside the home. In addition the concept of prohibitions can be complex and we only had data on whether a woman had a prohibition and not whether she agreed or disagreed with it.

## Conclusions

Given Nigeria’s diversity and large population, state-level analyses can provide valuable information to inform programs and policies at a more local level. Efforts to improve use of maternal and child health and family planning services in Plateau state, Nigeria, need to consider women’s empowerment, residence and poverty. Community education on the effectiveness of modern versus traditional methods and potential side effects of specific modern methods can help women make informed decisions on contraception. Understanding how best to provide services to the poorest and least empowered women is needed to ensure equitable access to maternal and child health services. Comprehensive efforts are also needed to address women’s empowerment, poverty reduction and education opportunities at the state and local levels.

## Supplementary Information

**Additional file 1 **: **Table S1.** Relative Risk Ratios (RRR) and 95% confidence intervals from logistic regression models for use of family planning methods and empowerment measures and Interactions after controlling for sociodemographic factors.

## Data Availability

The dataset supporting the conclusions of this article is available from the corresponding author and can be requested at this site: https://dataverse.unc.edu/dataverse/mle.

## Abbreviations

TFR: Total fertility rate
DPT: Diphtheria-pertussis-tetanus
IUD: Intra-uterine device
SDM: Standard days method
NURHI: Nigerian Urban Reproductive Health Initiative
UNFPA: United Nations Population Fund
