# Supplementary material for: The association of empowerment measures with maternal, child and family planning outcomes in Plateau State Nigeria by urban-rural residence
Source: BMC Pregnancy Childbirth. 2021 Feb 27;21:170. doi: 10.1186/s12884-021-03659-y (PMC7916291; doi:10.1186/s12884-021-03659-y)
Supplement: Supplementary file 1 — Additional file 1 : Table S1. Relative Risk Ratios (RRR) and 95% confidence intervals from logistic regression models for use of family planning methods and empowerment measures and Interactions after controlling for sociodemographic factors. [file 12884_2021_3659_MOESM1_ESM.docx]

Supplementary Table

| **Supplementary Table 1.** Relative Risk Ratios (RRR) and 95% confidence intervals from logistic regression models for use of family planning methods and empowerment measures and Interactions after controlling for sociodemographic factors. | | | |
| --- | --- | --- | --- |
| **Sociodemographic Factors** | **Traditional method vs**  **No Method** | **Modern method vs.**  **No method** | **Modern method vs. Traditional method** |
|  | **Urban** | **Urban Sample** | **Urban Sample** |
|  | **RRR (CI)** | **RRR [CI]** | **RRR [CI]** |
| **Age group** (Ref: 25-29) |  |  |  |
| 15-19 | 1.54 (0.14 - 16.65) | 2.00 (0.68 - 5.81) | 3.08 (0.30 - 31.44) |
| 20-24 | 0.46+ (0.21 - 1.01) | 1.36 (0.72 - 2.55) | 0.63 (0.27 - 1.46) |
| 30-34 | 0.84 (0.47 - 1.50) | 1.04 (0.65 - 1.66) | 0.87 (0.47 - 1.6) |
| 35+ | 1.53 (0.78 - 2.98) | 0.49** (0.32 - 0.75) | 0.75 (0.39 - 4.46) |
| **Parity** (Ref: 0-1) |  |  |  |
| 2 | 0.39* (0.18 - 0.82) | 3.68*** (2.28 - 6.94) | 1.44 (0.69 - 2.99) |
| 3-4 | 0.34** (0.16 - 0.72) | 7.06*** (4.47 - 11.15) | 2.45* (1.16 - 5.17) |
| 5+ | 0.34* (0.13 - 0.84) | 10.07*** (5.53 - 18.31) | 3.43** (1.38 - 8.51) |
| **Education level** (Ref: None/Non-standard) |  |  |  |
| Primary | 0.25** (0.09 - 0.69) | 0.85 (0.45 - 1.59) | 0.22** (0.08 - 0.58) |
| Secondary or higher | 0.26** (0.10 - 0.67) | 1.21 (0.65 - 2.24) | 0.32* (0.12 - 0.81) |
| **Wealth quintile** (Ref: Middle) |  |  |  |
| Lowest | 3.80* (1.23 - 11.74) | 0.87 (0.48 - 1.58) | 3.33* (1.01 - 10.95) |
| Second | 3.94** (1.50 - 10.31) | 0.63 (0.39 - 1.01) | 2.50+ (0.93 - 6.68 |
| Fourth | 1.61+ (0.95 - 2.72) | 0.82 (0.50 - 2.38) | 1.32 (0.73 - 2.38) |
| Highest | 1.14 (0.61 - 2.13) | 0.81 (0.54 - 1.72) | 0.93 (0.50 - 1.72) |
| **Religion** (Ref: Christian/Catholic) |  |  |  |
| Muslim/Other | 1.04 (0.59 - 1.83) | 0.18*** (0.11 - 0.36) | 1.18*** (0.09 - 0.36) |
| **Jos** (Ref: No) |  |  |  |
| Yes | 1.49 (0.30 – 7.19) | 2.23 (0.69 - 16.37) | 3.33 (0.67 - 16.37) |
| **Gender Equality Measures** |  |  |  |
| Household Decision Making | 0.83 (0.48 - 1.42) | 1.09 (0.83 - 1.42) | 0.91 (0.55 - 1.50) |
| Financial Decision-making | 1.52 (0.53 - 4.34) | 1.49 (0.79 - 2.82) | 2.29 (0.68 - 7.66) |
| Prohibitions | 0.19 (0.02 - 1.41) | 0.95 (0.33 - 2.73) | 0.18 (0.01 - 1.83) |
| Wife-beating Acceptable | 2.28** (1.29 - 4.04) | 1.48 (0.86 - 2.56) | 3.40*** (1.82 - 6.36) |
| **Interaction** |  |  |  |
| Household Decision Making X Jos | 1.01 (0.56 – 1.83) | 1.05 (0.79 – 1.39) | 1.07 (0.62 – 1.84) |
| Financial Decision-making X Jos | 0.31+ (0.09 – 1.00) | 0.72 (0.34 – 1.50) | 0.22* (0.05 – 0.85) |
| Prohibitions X Jos | 11.61* (1.38 – 97.36) | 0.50 (0.14 – 1.69) | 5.82 (0.49 – 68.71) |
| Wife-beating Acceptable X Jos | 0.70 (0.22 – 2.18) | 0.52+ (0.25 –1.06) | 0.36+ (0.11 – 1.16) |
| Note: The significance test is compared across the places of residence.  The unweighted count was 1192 for the urban sample. . | | | |
| +p<0.10 *p<0.05 **p<0.01 ***p<0.001 | | | |
